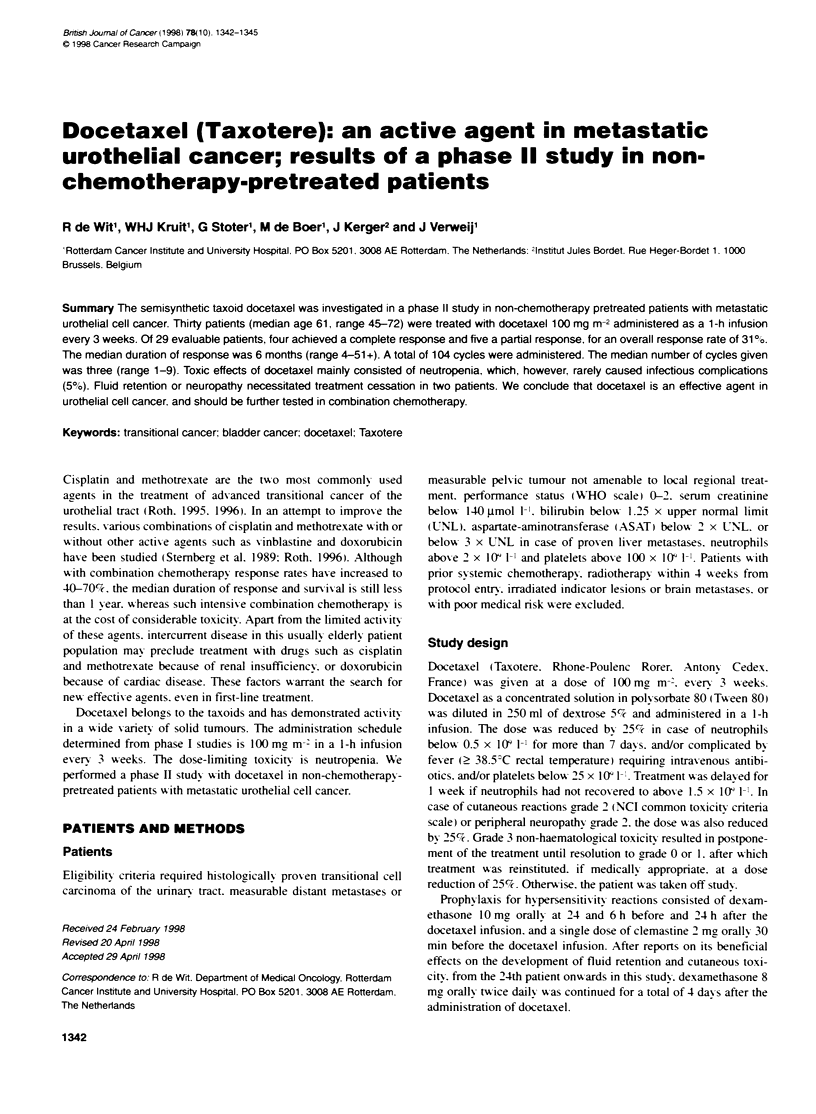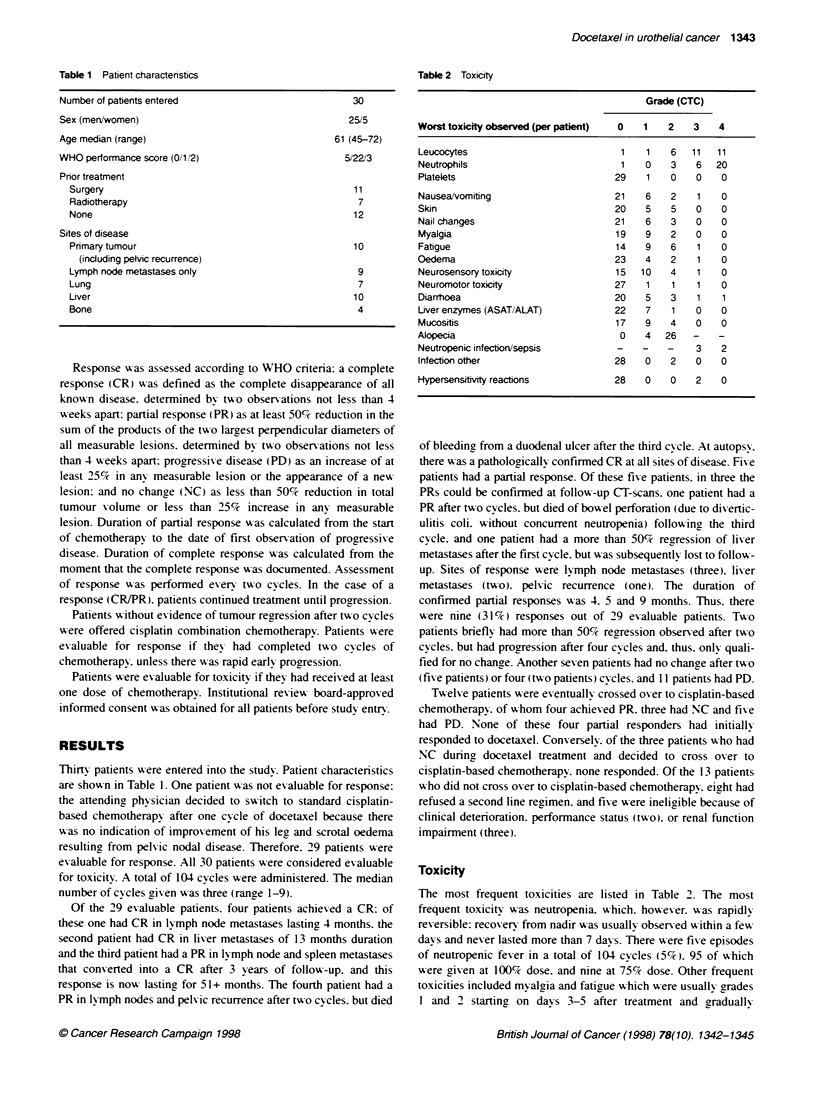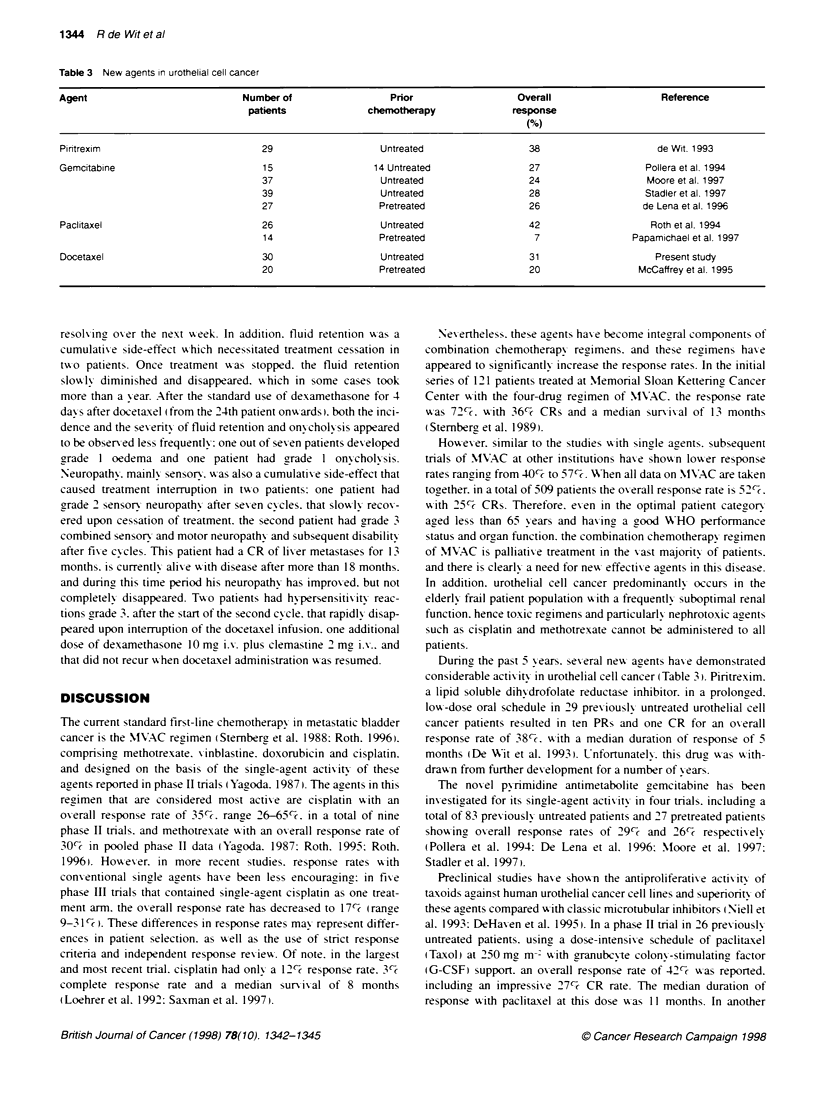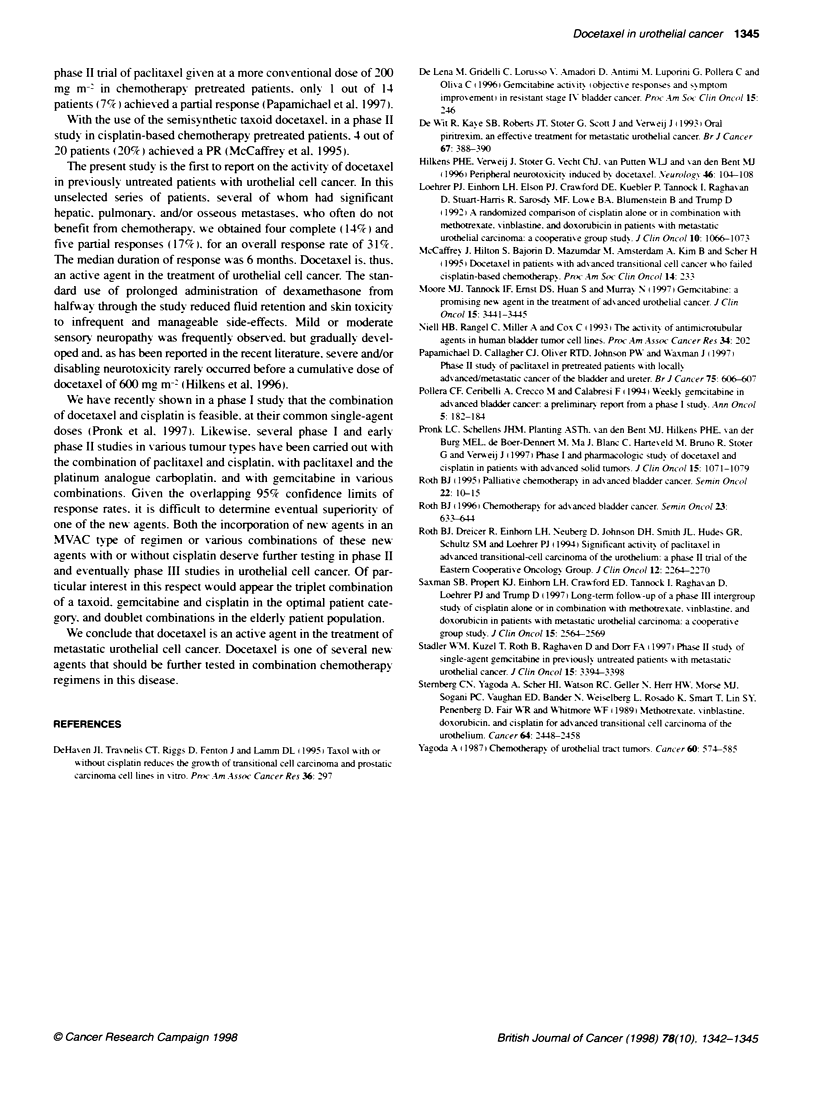# Docetaxel (Taxotere): an active agent in metastatic urothelial cancer; results of a phase II study in non-chemotherapy-pretreated patients.

**DOI:** 10.1038/bjc.1998.681

**Published:** 1998-11

**Authors:** R. de Wit, W. H. Kruit, G. Stoter, M. de Boer, J. Kerger, J. Verweij

**Affiliations:** Rotterdam Cancer Institute and University Hospital, The Netherlands.

## Abstract

The semisynthetic taxoid docetaxel was investigated in a phase II study in non-chemotherapy pretreated patients with metastatic urothelial cell cancer. Thirty patients (median age 61, range 45-72) were treated with docetaxel 100 mg m(-2) administered as a 1-h infusion every 3 weeks. Of 29 evaluable patients, four achieved a complete response and five a partial response, for an overall response rate of 31%. The median duration of response was 6 months (range 4-51+). A total of 104 cycles were administered. The median number of cycles given was three (range 1-9). Toxic effects of docetaxel mainly consisted of neutropenia, which, however, rarely caused infectious complications (5%). Fluid retention or neuropathy necessitated treatment cessation in two patients. We conclude that docetaxel is an effective agent in urothelial cell cancer, and should be further tested in combination chemotherapy.


British Journal of Cancer (1998) 78(10), 1342–1345
© 1998 Cancer Research Campaign

### Docetaxel (Taxotere): an active agent in metastatic urothelial cancer; results of a phase II study in non-chemotherapy-pretreated patients

**R de Wit[1], WHJ Kruit[1], G Stoter[1], M de Boer[1], J Kerger[2] and J Verweij[1]**

[1]Rotterdam Cancer Institute and University Hospital, PO Box 5201, 3008 AE Rotterdam, The Netherlands; [2]Institut Jules Bordet, Rue Heger-Bordet 1, 1000 Brussels, Belgium

**Summary** The semisynthetic taxoid docetaxel was investigated in a phase II study in non-chemotherapy pretreated patients with metastatic urothelial cell cancer. Thirty patients (median age 61, range 45–72) were treated with docetaxel 100 mg $m^{-2}$ administered as a 1-h infusion every 3 weeks. Of 29 evaluable patients, four achieved a complete response and five a partial response, for an overall response rate of 31%. The median duration of response was 6 months (range 4–51+). A total of 104 cycles were administered. The median number of cycles given was three (range 1–9). Toxic effects of docetaxel mainly consisted of neutropenia, which, however, rarely caused infectious complications (5%). Fluid retention or neuropathy necessitated treatment cessation in two patients. We conclude that docetaxel is an effective agent in urothelial cell cancer, and should be further tested in combination chemotherapy.

**Keywords:** transitional cancer; bladder cancer; docetaxel; Taxotere

Cisplatin and methotrexate are the two most commonly used agents in the treatment of advanced transitional cancer of the urothelial tract (Roth, 1995, 1996). In an attempt to improve the results, various combinations of cisplatin and methotrexate with or without other active agents such as vinblastine and doxorubicin have been studied (Sternberg et al, 1989; Roth, 1996). Although with combination chemotherapy response rates have increased to 40–70%, the median duration of response and survival is still less than 1 year, whereas such intensive combination chemotherapy is at the cost of considerable toxicity. Apart from the limited activity of these agents, intercurrent disease in this usually elderly patient population may preclude treatment with drugs such as cisplatin and methotrexate because of renal insufficiency, or doxorubicin because of cardiac disease. These factors warrant the search for new effective agents, even in first-line treatment.

Docetaxel belongs to the taxoids and has demonstrated activity in a wide variety of solid tumours. The administration schedule determined from phase I studies is 100 mg $m^{-2}$ in a 1-h infusion every 3 weeks. The dose-limiting toxicity is neutropenia. We performed a phase II study with docetaxel in non-chemotherapy-pretreated patients with metastatic urothelial cell cancer.

### PATIENTS AND METHODS

### Patients

Eligibility criteria required histologically proven transitional cell carcinoma of the urinary tract, measurable distant metastases or measurable pelvic tumour not amenable to local regional treatment, performance status (WHO scale) 0–2, serum creatinine below 140 µmol $l^{-1}$, bilirubin below 1.25 × upper normal limit (UNL), aspartate-aminotransferase (ASAT) below 2 × UNL, or below 3 × UNL in case of proven liver metastases, neutrophils above $2 \times 10^9$ $l^{-1}$ and platelets above $100 \times 10^9$ $l^{-1}$. Patients with prior systemic chemotherapy, radiotherapy within 4 weeks from protocol entry, irradiated indicator lesions or brain metastases, or with poor medical risk were excluded.

*Received 24 February 1998*
*Revised 20 April 1998*
*Accepted 29 April 1998*

*Correspondence to:* R de Wit, Department of Medical Oncology, Rotterdam Cancer Institute and University Hospital, PO Box 5201, 3008 AE Rotterdam, The Netherlands

### Study design

Docetaxel (Taxotere, Rhone-Poulenc Rorer, Antony Cedex, France) was given at a dose of 100 mg $m^{-2}$, every 3 weeks. Docetaxel as a concentrated solution in polysorbate 80 (Tween 80) was diluted in 250 ml of dextrose 5% and administered in a 1-h infusion. The dose was reduced by 25% in case of neutrophils below $0.5 \times 10^9$ $l^{-1}$ for more than 7 days, and/or complicated by fever (≥ 38.5°C rectal temperature) requiring intravenous antibiotics, and/or platelets below $25 \times 10^9$ $l^{-1}$. Treatment was delayed for 1 week if neutrophils had not recovered to above $1.5 \times 10^9$ $l^{-1}$. In case of cutaneous reactions grade 2 (NCI common toxicity criteria scale) or peripheral neuropathy grade 2, the dose was also reduced by 25%. Grade 3 non-haematological toxicity resulted in postponement of the treatment until resolution to grade 0 or 1, after which treatment was reinstituted, if medically appropriate, at a dose reduction of 25%. Otherwise, the patient was taken off study.

Prophylaxis for hypersensitivity reactions consisted of dexamethasone 10 mg orally at 24 and 6 h before and 24 h after the docetaxel infusion, and a single dose of clemastine 2 mg orally 30 min before the docetaxel infusion. After reports on its beneficial effects on the development of fluid retention and cutaneous toxicity, from the 24th patient onwards in this study, dexamethasone 8 mg orally twice daily was continued for a total of 4 days after the administration of docetaxel.

**Table 1** Patient characteristics

| | |
|---|---|
| Number of patients entered | 30 |
| Sex (men/women) | 25/5 |
| Age median (range) | 61 (45–72) |
| WHO performance score (0/1/2) | 5/22/3 |
| Prior treatment | |
| Surgery | 11 |
| Radiotherapy | 7 |
| None | 12 |
| Sites of disease | |
| Primary tumour (including pelvic recurrence) | 10 |
| Lymph node metastases only | 9 |
| Lung | 7 |
| Liver | 10 |
| Bone | 4 |

**Table 2** Toxicity

| | Grade (CTC) | | | | |
|---|---|---|---|---|---|
| **Worst toxicity observed (per patient)** | **0** | **1** | **2** | **3** | **4** |
| Leucocytes | 1 | 1 | 6 | 11 | 11 |
| Neutrophils | 1 | 0 | 3 | 6 | 20 |
| Platelets | 29 | 1 | 0 | 0 | 0 |
| Nausea/vomiting | 21 | 6 | 2 | 1 | 0 |
| Skin | 20 | 5 | 5 | 0 | 0 |
| Nail changes | 21 | 6 | 3 | 0 | 0 |
| Myalgia | 19 | 9 | 2 | 0 | 0 |
| Fatigue | 14 | 9 | 6 | 1 | 0 |
| Oedema | 23 | 4 | 2 | 1 | 0 |
| Neurosensory toxicity | 15 | 10 | 4 | 1 | 0 |
| Neuromotor toxicity | 27 | 1 | 1 | 1 | 0 |
| Diarrhoea | 20 | 5 | 3 | 1 | 1 |
| Liver enzymes (ASAT/ALAT) | 22 | 7 | 1 | 0 | 0 |
| Mucositis | 17 | 9 | 4 | 0 | 0 |
| Alopecia | 0 | 4 | 26 | – | – |
| Neutropenic infection/sepsis | – | – | – | 3 | 2 |
| Infection other | 28 | 0 | 2 | 0 | 0 |
| Hypersensitivity reactions | 28 | 0 | 0 | 2 | 0 |

Response was assessed according to WHO criteria: a complete response (CR) was defined as the complete disappearance of all known disease, determined by two observations not less than 4 weeks apart; partial response (PR) as at least 50% reduction in the sum of the products of the two largest perpendicular diameters of all measurable lesions, determined by two observations not less than 4 weeks apart; progressive disease (PD) as an increase of at least 25% in any measurable lesion or the appearance of a new lesion; and no change (NC) as less than 50% reduction in total tumour volume or less than 25% increase in any measurable lesion. Duration of partial response was calculated from the start of chemotherapy to the date of first observation of progressive disease. Duration of complete response was calculated from the moment that the complete response was documented. Assessment of response was performed every two cycles. In the case of a response (CR/PR), patients continued treatment until progression.

Patients without evidence of tumour regression after two cycles were offered cisplatin combination chemotherapy. Patients were evaluable for response if they had completed two cycles of chemotherapy, unless there was rapid early progression.

Patients were evaluable for toxicity if they had received at least one dose of chemotherapy. Institutional review board-approved informed consent was obtained for all patients before study entry.

### RESULTS

Thirty patients were entered into the study. Patient characteristics are shown in Table 1. One patient was not evaluable for response; the attending physician decided to switch to standard cisplatin-based chemotherapy after one cycle of docetaxel because there was no indication of improvement of his leg and scrotal oedema resulting from pelvic nodal disease. Therefore, 29 patients were evaluable for response. All 30 patients were considered evaluable for toxicity. A total of 104 cycles were administered. The median number of cycles given was three (range 1–9).

Of the 29 evaluable patients, four patients achieved a CR; of these one had CR in lymph node metastases lasting 4 months, the second patient had CR in liver metastases of 13 months duration and the third patient had a PR in lymph node and spleen metastases that converted into a CR after 3 years of follow-up, and this response is now lasting for 51+ months. The fourth patient had a PR in lymph nodes and pelvic recurrence after two cycles, but died of bleeding from a duodenal ulcer after the third cycle. At autopsy, there was a pathologically confirmed CR at all sites of disease. Five patients had a partial response. Of these five patients, in three the PRs could be confirmed at follow-up CT-scans, one patient had a PR after two cycles, but died of bowel perforation (due to diverticulitis coli, without concurrent neutropenia) following the third cycle, and one patient had a more than 50% regression of liver metastases after the first cycle, but was subsequently lost to follow-up. Sites of response were lymph node metastases (three), liver metastases (two), pelvic recurrence (one). The duration of confirmed partial responses was 4, 5 and 9 months. Thus, there were nine (31%) responses out of 29 evaluable patients. Two patients briefly had more than 50% regression observed after two cycles, but had progression after four cycles and, thus, only qualified for no change. Another seven patients had no change after two (five patients) or four (two patients) cycles, and 11 patients had PD.

Twelve patients were eventually crossed over to cisplatin-based chemotherapy, of whom four achieved PR, three had NC and five had PD. None of these four partial responders had initially responded to docetaxel. Conversely, of the three patients who had NC during docetaxel treatment and decided to cross over to cisplatin-based chemotherapy, none responded. Of the 13 patients who did not cross over to cisplatin-based chemotherapy, eight had refused a second line regimen, and five were ineligible because of clinical deterioration, performance status (two), or renal function impairment (three).

### Toxicity

The most frequent toxicities are listed in Table 2. The most frequent toxicity was neutropenia, which, however, was rapidly reversible; recovery from nadir was usually observed within a few days and never lasted more than 7 days. There were five episodes of neutropenic fever in a total of 104 cycles (5%), 95 of which were given at 100% dose, and nine at 75% dose. Other frequent toxicities included myalgia and fatigue which were usually grades 1 and 2 starting on days 3–5 after treatment and gradually

© Cancer Research Campaign 1998

**Table 3** New agents in urothelial cell cancer

| Agent | Number of patients | Prior chemotherapy | Overall response (%) | Reference |
|---|---|---|---|---|
| Piritrexim | 29 | Untreated | 38 | de Wit. 1993 |
| Gemcitabine | 15 | 14 Untreated | 27 | Pollera et al. 1994 |
| | 37 | Untreated | 24 | Moore et al. 1997 |
| | 39 | Untreated | 28 | Stadler et al. 1997 |
| | 27 | Pretreated | 26 | de Lena et al. 1996 |
| Paclitaxel | 26 | Untreated | 42 | Roth et al. 1994 |
| | 14 | Pretreated | 7 | Papamichael et al. 1997 |
| Docetaxel | 30 | Untreated | 31 | Present study |
| | 20 | Pretreated | 20 | McCaffrey et al. 1995 |

resolving over the next week. In addition, fluid retention was a cumulative side-effect which necessitated treatment cessation in two patients. Once treatment was stopped, the fluid retention slowly diminished and disappeared, which in some cases took more than a year. After the standard use of dexamethasone for 4 days after docetaxel (from the 24th patient onwards), both the incidence and the severity of fluid retention and onycholysis appeared to be observed less frequently: one out of seven patients developed grade 1 oedema and one patient had grade 1 onycholysis. Neuropathy, mainly sensory, was also a cumulative side-effect that caused treatment interruption in two patients: one patient had grade 2 sensory neuropathy after seven cycles, that slowly recovered upon cessation of treatment, the second patient had grade 3 combined sensory and motor neuropathy and subsequent disability after five cycles. This patient had a CR of liver metastases for 13 months, is currently alive with disease after more than 18 months, and during this time period his neuropathy has improved, but not completely disappeared. Two patients had hypersensitivity reactions grade 3, after the start of the second cycle, that rapidly disappeared upon interruption of the docetaxel infusion, one additional dose of dexamethasone 10 mg i.v. plus clemastine 2 mg i.v., and that did not recur when docetaxel administration was resumed.

### DISCUSSION

The current standard first-line chemotherapy in metastatic bladder cancer is the MVAC regimen (Sternberg et al. 1988; Roth, 1996), comprising methotrexate, vinblastine, doxorubicin and cisplatin, and designed on the basis of the single-agent activity of these agents reported in phase II trials (Yagoda, 1987). The agents in this regimen that are considered most active are cisplatin with an overall response rate of 35%, range 26–65%, in a total of nine phase II trials, and methotrexate with an overall response rate of 30% in pooled phase II data (Yagoda, 1987; Roth, 1995; Roth, 1996). However, in more recent studies, response rates with conventional single agents have been less encouraging: in five phase III trials that contained single-agent cisplatin as one treatment arm, the overall response rate has decreased to 17% (range 9–31%). These differences in response rates may represent differences in patient selection, as well as the use of strict response criteria and independent response review. Of note, in the largest and most recent trial, cisplatin had only a 12% response rate, 3% complete response rate and a median survival of 8 months (Loehrer et al. 1992; Saxman et al. 1997).

Nevertheless, these agents have become integral components of combination chemotherapy regimens, and these regimens have appeared to significantly increase the response rates. In the initial series of 121 patients treated at Memorial Sloan Kettering Cancer Center with the four-drug regimen of MVAC, the response rate was 72%, with 36% CRs and a median survival of 13 months (Sternberg et al. 1989).

However, similar to the studies with single agents, subsequent trials of MVAC at other institutions have shown lower response rates ranging from 40% to 57%. When all data on MVAC are taken together, in a total of 509 patients the overall response rate is 52%, with 25% CRs. Therefore, even in the optimal patient category aged less than 65 years and having a good WHO performance status and organ function, the combination chemotherapy regimen of MVAC is palliative treatment in the vast majority of patients, and there is clearly a need for new effective agents in this disease. In addition, urothelial cell cancer predominantly occurs in the elderly frail patient population with a frequently suboptimal renal function, hence toxic regimens and particularly nephrotoxic agents such as cisplatin and methotrexate cannot be administered to all patients.

During the past 5 years, several new agents have demonstrated considerable activity in urothelial cell cancer (Table 3). Piritrexim, a lipid soluble dihydrofolate reductase inhibitor, in a prolonged, low-dose oral schedule in 29 previously untreated urothelial cell cancer patients resulted in ten PRs and one CR for an overall response rate of 38%, with a median duration of response of 5 months (De Wit et al. 1993). Unfortunately, this drug was withdrawn from further development for a number of years.

The novel pyrimidine antimetabolite gemcitabine has been investigated for its single-agent activity in four trials, including a total of 83 previously untreated patients and 27 pretreated patients showing overall response rates of 29% and 26% respectively (Pollera et al. 1994; De Lena et al. 1996; Moore et al. 1997; Stadler et al. 1997).

Preclinical studies have shown the antiproliferative activity of taxoids against human urothelial cancer cell lines and superiority of these agents compared with classic microtubular inhibitors (Niell et al. 1993; DeHaven et al. 1995). In a phase II trial in 26 previously untreated patients, using a dose-intensive schedule of paclitaxel (Taxol) at 250 mg $m^{-2}$ with granubcyte colony-stimulating factor (G-CSF) support, an overall response rate of 42% was reported, including an impressive 27% CR rate. The median duration of response with paclitaxel at this dose was 11 months. In another

 © Cancer Research Campaign 1998

phase II trial of paclitaxel given at a more conventional dose of 200 mg m$^{-2}$ in chemotherapy pretreated patients, only 1 out of 14 patients (7%) achieved a partial response (Papamichael et al, 1997).

With the use of the semisynthetic taxoid docetaxel, in a phase II study in cisplatin-based chemotherapy pretreated patients, 4 out of 20 patients (20%) achieved a PR (McCaffrey et al, 1995).

The present study is the first to report on the activity of docetaxel in previously untreated patients with urothelial cell cancer. In this unselected series of patients, several of whom had significant hepatic, pulmonary, and/or osseous metastases, who often do not benefit from chemotherapy, we obtained four complete (14%) and five partial responses (17%), for an overall response rate of 31%. The median duration of response was 6 months. Docetaxel is, thus, an active agent in the treatment of urothelial cell cancer. The standard use of prolonged administration of dexamethasone from halfway through the study reduced fluid retention and skin toxicity to infrequent and manageable side-effects. Mild or moderate sensory neuropathy was frequently observed, but gradually developed and, as has been reported in the recent literature, severe and/or disabling neurotoxicity rarely occurred before a cumulative dose of docetaxel of 600 mg m$^{-2}$ (Hilkens et al, 1996).

We have recently shown in a phase I study that the combination of docetaxel and cisplatin is feasible, at their common single-agent doses (Pronk et al, 1997). Likewise, several phase I and early phase II studies in various tumour types have been carried out with the combination of paclitaxel and cisplatin, with paclitaxel and the platinum analogue carboplatin, and with gemcitabine in various combinations. Given the overlapping 95% confidence limits of response rates, it is difficult to determine eventual superiority of one of the new agents. Both the incorporation of new agents in an MVAC type of regimen or various combinations of these new agents with or without cisplatin deserve further testing in phase II and eventually phase III studies in urothelial cell cancer. Of particular interest in this respect would appear the triplet combination of a taxoid, gemcitabine and cisplatin in the optimal patient category, and doublet combinations in the elderly patient population.

We conclude that docetaxel is an active agent in the treatment of metastatic urothelial cell cancer. Docetaxel is one of several new agents that should be further tested in combination chemotherapy regimens in this disease.

### REFERENCES

DeHaven JI, Traynelis CT, Riggs D, Fenton J and Lamm DL (1995) Taxol with or without cisplatin reduces the growth of transitional cell carcinoma and prostatic carcinoma cell lines in vitro. *Proc Am Assoc Cancer Res* **36**: 297

De Lena M, Gridelli C, Lorusso V, Amadori D, Antimi M, Luporini G, Pollera C and Oliva C (1996) Gemcitabine activity (objective responses and symptom improvement) in resistant stage IV bladder cancer. *Proc Am Soc Clin Oncol* **15**: 246

De Wit R, Kaye SB, Roberts JT, Stoter G, Scott J and Verweij J (1993) Oral piritrexim, an effective treatment for metastatic urothelial cancer. *Br J Cancer* **67**: 388–390

Hilkens PHE, Verweij J, Stoter G, Vecht ChJ, van Putten WLJ and van den Bent MJ (1996) Peripheral neurotoxicity induced by docetaxel. *Neurology* **46**: 104–108

Loehrer PJ, Einhorn LH, Elson PJ, Crawford DE, Kuebler P, Tannock I, Raghavan D, Stuart-Harris R, Sarosdy MF, Lowe BA, Blumenstein B and Trump D (1992) A randomized comparison of cisplatin alone or in combination with methotrexate, vinblastine, and doxorubicin in patients with metastatic urothelial carcinoma: a cooperative group study. *J Clin Oncol* **10**: 1066–1073

McCaffrey J, Hilton S, Bajorin D, Mazumdar M, Amsterdam A, Kim B and Scher H (1995) Docetaxel in patients with advanced transitional cell cancer who failed cisplatin-based chemotherapy. *Proc Am Soc Clin Oncol* **14**: 233

Moore MJ, Tannock IF, Ernst DS, Huan S and Murray N (1997) Gemcitabine: a promising new agent in the treatment of advanced urothelial cancer. *J Clin Oncol* **15**: 3441–3445

Niell HB, Rangel C, Miller A and Cox C (1993) The activity of antimicrotubular agents in human bladder tumor cell lines. *Proc Am Assoc Cancer Res* **34**: 202

Papamichael D, Callagher CJ, Oliver RTD, Johnson PW and Waxman J (1997) Phase II study of paclitaxel in pretreated patients with locally advanced/metastatic cancer of the bladder and ureter. *Br J Cancer* **75**: 606–607

Pollera CF, Ceribelli A, Crecco M and Calabresi F (1994) Weekly gemcitabine in advanced bladder cancer: a preliminary report from a phase I study. *Ann Oncol* **5**: 182–184

Pronk LC, Schellens JHM, Planting ASTh, van den Bent MJ, Hilkens PHE, van der Burg MEL, de Boer-Dennert M, Ma J, Blanc C, Harteveld M, Bruno R, Stoter G and Verweij J (1997) Phase I and pharmacologic study of docetaxel and cisplatin in patients with advanced solid tumors. *J Clin Oncol* **15**: 1071–1079

Roth BJ (1995) Palliative chemotherapy in advanced bladder cancer. *Semin Oncol* **22**: 10–15

Roth BJ (1996) Chemotherapy for advanced bladder cancer. *Semin Oncol* **23**: 633–644

Roth BJ, Dreicer R, Einhorn LH, Neuberg D, Johnson DH, Smith JL, Hudes GR, Schultz SM and Loehrer PJ (1994) Significant activity of paclitaxel in advanced transitional-cell carcinoma of the urothelium: a phase II trial of the Eastern Cooperative Oncology Group. *J Clin Oncol* **12**: 2264–2270

Saxman SB, Propert KJ, Einhorn LH, Crawford ED, Tannock I, Raghavan D, Loehrer PJ and Trump D (1997) Long-term follow-up of a phase III intergroup study of cisplatin alone or in combination with methotrexate, vinblastine, and doxorubicin in patients with metastatic urothelial carcinoma: a cooperative group study. *J Clin Oncol* **15**: 2564–2569

Stadler WM, Kuzel T, Roth B, Raghaven D and Dorr FA (1997) Phase II study of single-agent gemcitabine in previously untreated patients with metastatic urothelial cancer. *J Clin Oncol* **15**: 3394–3398

Sternberg CN, Yagoda A, Scher HI, Watson RC, Geller N, Herr HW, Morse MJ, Sogani PC, Vaughan ED, Bander N, Weiselberg L, Rosado K, Smart T, Lin SY, Penenberg D, Fair WR and Whitmore WF (1989) Methotrexate, vinblastine, doxorubicin, and cisplatin for advanced transitional cell carcinoma of the urothelium. *Cancer* **64**: 2448–2458

Yagoda A (1987) Chemotherapy of urothelial tract tumors. *Cancer* **60**: 574–585

© Cancer Research Campaign 1998